# Report of Deaths in Buffalo for the Month of December, 1855

**Published:** 1856-02

**Authors:** J. Root

**Affiliations:** Health Physician


### ART. VII.—*Report of Deaths in Buffalo for the month*

| DISEASES. | Males. | Females. | Total. | AGE. Under 1 year. Male. | Under 1 year. Female. | 1 to 2 years. Male. | 1 to 2 years. Female. |
|---|---|---|---|---|---|---|---|
| Apoplexy, | 2 | | 2 | | | | |
| Asthma, | 1 | | 1 | | | | |
| Bronchial Catarrh, | 1 | | 1 | 1 | | | |
| Consumption, | 7 | 5 | 12 | 1 | 1 | 1 | |
| Convulsions, | 4 | 1 | 5 | 3 | | 1 | 1 |
| Cancer, | 1 | | 1 | | | | |
| Child Bed, | | 1 | 1 | | | | |
| Croup, | 2 | 1 | 3 | | | | 1 |
| Croup Membranous, | 1 | | 1 | | | | |
| Congestion of Lungs, | 1 | 1 | 2 | 1 | 1 | | |
| Cerebro-spinal Meningitis, | 1 | | 1 | | | | |
| Drowned, | 1 | | 1 | | | | |
| Diarrhœa, | 1 | | 1 | | | | |
| Debility, | 1 | 1 | 2 | 1 | 1 | | |
| Dentition, | | 1 | 1 | | | | |
| Dropsy, | 2 | 1 | 3 | 1 | | | |
| Dropsy of Brain, | 1 | | 1 | | | 1 | |
| Delirium Tremens, | 1 | | 1 | | | | |
| Epilepsy, | 1 | | 1 | | | | |
| Fever, | 2 | 1 | 3 | | 1 | | |
| " Bilious, | 1 | 1 | 2 | | | | |
| " Typhus, | 1 | 2 | 3 | | | | |
| " Typhoid, | 1 | 2 | 3 | | | | |
| " Scarlet, | 7 | 4 | 11 | | | | 2 |
| Gastro-enteritis, | 1 | | 1 | | | | |
| Hydrocephalus, | 2 | | 2 | | | 2 | |
| Heart Disease, | 1 | | 1 | | | | |
| Inflammation, | 1 | 1 | 2 | | | | |
| Inflammation of Bowels, | 1 | | 1 | 1 | | | |
| Inflamm–tion of Brain, | | 2 | 2 | | 1 | | |
| Intemperance, | 1 | | 1 | | | | |
| Marasmus, | 1 | 1 | 2 | 1 | | | 1 |
| Old Age, | 3 | 1 | 4 | | | | |
| Peritonitis, | 2 | | 2 | 1 | | | |
| Paralysis, | 1 | 2 | 3 | | | | |
| Pneumonia, | 3 | 1 | 4 | 2 | | | |
| Still-born, | 2 | 2 | 4 | 2 | 2 | | |
| Small-pox, | 1 | | 1 | | | | |
| Unknown, | 8 | 2 | 10 | 4 | 2 | | |
| Vomiting, | 1 | | 1 | | | | |
| Whooping Cough, | 1 | 3 | 4 | | 1 | | 1 |
| Totals, | 71 | 37 | 108 | 19 | 10 | 5 | 6 |

NATIVITIES.—American, 57. German, 27. Irish, 8. French, 5.

*of December*, 18 55. By J. Root, M. D., Health Physician.

AGE.

| 2—5 | | 5—10 | | 10—15 | | 15—20 | | 20—25 | | 25—30 | | 30—40 | | 40—50 | | 50—60 | | 60—70 | | 70—80 | | 80—90 | | 90—100 | | 100 and upward. | | Not given. | |
|---|---|---|---|---|---|---|---|---|---|---|---|---|---|---|---|---|---|---|---|---|---|---|---|---|---|---|---|---|---|
| Male. | Female. | Male. | Female. | Male. | Female. | Male. | Female. | Male. | Female. | Male. | Female. | Male. | Female. | Male. | Female. | Male. | Female. | Male. | Female. | Male. | Female. | Male. | Female. | Male. | Female. | Male. | Female. | Male. | Female. |
| -- | -- | -- | -- | -- | -- | -- | -- | -- | -- | -- | -- | -- | -- | -- | -- | -- | -- | 1 | -- | 1 | -- | -- | -- | -- | -- | -- | -- | -- | -- |
| -- | -- | 1 | -- | -- | -- | -- | -- | -- | -- | -- | -- | -- | -- | -- | -- | -- | -- | -- | -- | -- | -- | -- | -- | -- | -- | -- | -- | -- | -- |
| -- | -- | -- | -- | -- | -- | -- | -- | -- | -- | -- | -- | -- | -- | -- | -- | -- | -- | -- | -- | -- | -- | -- | -- | -- | -- | -- | -- | -- | -- |
| -- | -- | 1 | -- | -- | 1 | -- | -- | -- | -- | 1 | -- | 3 | -- | -- | -- | -- | 2 | -- | 1 | -- | -- | -- | -- | -- | -- | -- | -- | -- | -- |
| -- | -- | -- | -- | -- | -- | -- | -- | -- | -- | -- | -- | -- | -- | -- | -- | -- | -- | -- | -- | -- | -- | -- | -- | -- | -- | -- | -- | -- | -- |
| -- | -- | -- | -- | -- | -- | -- | -- | -- | -- | -- | -- | 1 | -- | -- | -- | -- | -- | -- | -- | -- | -- | -- | -- | -- | -- | -- | -- | -- | -- |
| -- | -- | -- | -- | -- | -- | -- | -- | -- | -- | -- | -- | -- | -- | -- | 1 | -- | -- | -- | -- | -- | -- | -- | -- | -- | -- | -- | -- | -- | -- |
| 2 | -- | -- | -- | -- | -- | -- | -- | -- | -- | -- | -- | -- | -- | -- | -- | -- | -- | -- | -- | -- | -- | -- | -- | -- | -- | -- | -- | -- | -- |
| 1 | -- | -- | -- | -- | -- | -- | -- | -- | -- | -- | -- | -- | -- | -- | -- | -- | -- | -- | -- | -- | -- | -- | -- | -- | -- | -- | -- | -- | -- |
| -- | -- | -- | -- | -- | -- | -- | -- | -- | -- | -- | -- | -- | -- | -- | -- | -- | -- | -- | -- | -- | -- | -- | -- | -- | -- | -- | -- | -- | -- |
| 1 | -- | -- | -- | -- | -- | -- | -- | -- | -- | -- | -- | -- | -- | -- | -- | -- | -- | -- | -- | -- | -- | -- | -- | -- | -- | -- | -- | -- | -- |
| -- | -- | -- | -- | -- | -- | -- | -- | 1 | -- | -- | -- | -- | -- | -- | -- | -- | -- | -- | -- | -- | -- | -- | -- | -- | -- | -- | -- | -- | -- |
| -- | -- | -- | -- | -- | -- | -- | -- | -- | -- | *1* | -- | -- | -- | -- | -- | -- | -- | -- | -- | -- | -- | -- | -- | -- | -- | -- | -- | -- | -- |
| -- | -- | -- | -- | -- | -- | -- | -- | -- | -- | -- | -- | -- | -- | -- | -- | -- | -- | -- | -- | -- | -- | -- | -- | -- | -- | -- | -- | -- | -- |
| -- | 1 | -- | -- | -- | -- | -- | -- | -- | -- | -- | -- | -- | -- | -- | -- | -- | -- | -- | -- | -- | -- | -- | -- | -- | -- | -- | -- | -- | -- |
| -- | -- | -- | -- | -- | -- | -- | -- | -- | -- | -- | 1 | 1 | -- | -- | -- | -- | -- | -- | -- | -- | -- | -- | -- | -- | -- | -- | -- | -- | -- |
| -- | -- | -- | -- | -- | -- | -- | -- | -- | -- | -- | -- | -- | -- | -- | -- | -- | -- | -- | -- | -- | -- | -- | -- | -- | -- | -- | -- | -- | -- |
| -- | -- | -- | -- | -- | -- | -- | -- | -- | -- | -- | -- | -- | -- | -- | -- | 1 | -- | -- | -- | -- | -- | -- | -- | -- | -- | -- | -- | -- | -- |
| -- | -- | -- | -- | -- | -- | -- | -- | 1 | -- | -- | -- | -- | -- | -- | -- | -- | -- | -- | -- | -- | -- | -- | -- | -- | -- | -- | -- | -- | -- |
| 1 | -- | 1 | -- | -- | -- | -- | -- | -- | -- | -- | -- | -- | -- | -- | -- | -- | -- | -- | -- | -- | -- | -- | -- | -- | -- | -- | -- | -- | -- |
| -- | -- | -- | -- | -- | -- | -- | -- | -- | -- | -- | -- | 1 | -- | -- | -- | -- | -- | -- | 1 | -- | -- | -- | -- | -- | -- | -- | -- | -- | -- |
| -- | -- | -- | 2 | -- | -- | -- | -- | -- | -- | -- | -- | 1 | -- | -- | -- | -- | -- | -- | -- | -- | -- | -- | -- | -- | -- | -- | -- | -- | -- |
| -- | 1 | -- | -- | -- | -- | -- | -- | -- | -- | -- | -- | 1 | 1 | -- | -- | -- | -- | -- | -- | -- | -- | -- | -- | -- | -- | -- | -- | -- | -- |
| 7 | -- | -- | 2 | -- | -- | -- | -- | -- | -- | -- | -- | -- | -- | -- | -- | -- | -- | -- | -- | -- | -- | -- | -- | -- | -- | -- | -- | -- | -- |
| -- | -- | -- | -- | -- | -- | -- | -- | -- | -- | -- | -- | -- | -- | -- | -- | 1 | -- | -- | -- | -- | -- | -- | -- | -- | -- | -- | -- | -- | -- |
| -- | -- | -- | -- | -- | -- | -- | -- | -- | -- | -- | -- | -- | -- | -- | -- | -- | -- | -- | -- | -- | -- | -- | -- | -- | -- | -- | -- | -- | -- |
| -- | -- | -- | -- | -- | -- | -- | -- | -- | -- | -- | -- | -- | -- | -- | -- | -- | -- | 1 | -- | -- | -- | -- | -- | -- | -- | -- | -- | -- | -- |
| -- | 1 | -- | -- | -- | -- | -- | -- | -- | -- | -- | -- | -- | -- | -- | -- | 1 | -- | -- | -- | -- | -- | -- | -- | -- | -- | -- | -- | -- | -- |
| -- | -- | -- | -- | -- | -- | -- | -- | -- | -- | -- | -- | -- | -- | -- | -- | -- | -- | -- | -- | -- | -- | -- | -- | -- | -- | -- | -- | -- | -- |
| -- | 1 | -- | -- | -- | -- | -- | -- | -- | -- | -- | -- | -- | -- | -- | -- | -- | -- | -- | -- | -- | -- | -- | -- | -- | -- | -- | -- | -- | -- |
| -- | -- | -- | -- | -- | -- | -- | -- | -- | -- | -- | -- | -- | -- | 1 | -- | -- | -- | -- | -- | -- | -- | -- | -- | -- | -- | -- | -- | -- | -- |
| -- | -- | -- | -- | -- | -- | -- | -- | -- | -- | -- | -- | -- | -- | -- | -- | -- | -- | -- | -- | -- | -- | -- | -- | -- | -- | -- | -- | -- | -- |
| -- | -- | -- | -- | -- | -- | -- | -- | -- | -- | -- | -- | -- | -- | -- | -- | -- | -- | -- | -- | 1 | 1 | 2 | -- | -- | -- | -- | -- | -- | -- |
| -- | -- | -- | -- | -- | -- | -- | -- | -- | -- | -- | -- | 1 | -- | -- | -- | -- | -- | -- | -- | -- | -- | -- | -- | -- | -- | -- | -- | -- | -- |
| -- | -- | -- | -- | -- | -- | -- | -- | -- | 1 | -- | -- | -- | -- | -- | -- | -- | -- | -- | 1 | -- | -- | 1 | -- | -- | -- | -- | -- | -- | -- |
| -- | -- | -- | -- | -- | -- | -- | -- | -- | -- | -- | -- | -- | -- | -- | -- | -- | -- | 1 | -- | -- | 1 | -- | -- | -- | -- | -- | -- | -- | -- |
| -- | -- | -- | -- | -- | -- | -- | -- | -- | -- | -- | -- | -- | -- | -- | -- | -- | -- | -- | -- | -- | -- | -- | -- | -- | -- | -- | -- | -- | -- |
| -- | -- | -- | -- | -- | -- | -- | -- | 1 | -- | -- | -- | -- | -- | -- | -- | -- | -- | -- | -- | -- | -- | -- | -- | -- | -- | -- | -- | -- | -- |
| 1 | -- | -- | -- | 1 | -- | -- | -- | -- | -- | -- | -- | 1 | -- | 1 | -- | -- | -- | -- | -- | -- | -- | -- | -- | -- | -- | -- | -- | -- | -- |
| 1 | -- | -- | -- | -- | -- | -- | -- | -- | -- | -- | -- | -- | -- | -- | -- | -- | -- | -- | -- | -- | -- | -- | -- | -- | -- | -- | -- | -- | -- |
| 1 | 1 | -- | -- | -- | -- | -- | -- | -- | -- | -- | -- | -- | -- | -- | -- | -- | -- | -- | -- | -- | -- | -- | -- | -- | -- | -- | -- | -- | -- |
| -- | -- | -- | -- | -- | -- | -- | -- | -- | -- | -- | -- | -- | -- | -- | -- | -- | -- | -- | -- | -- | -- | -- | -- | -- | -- | -- | -- | -- | -- |
| -- | -- | -- | -- | -- | -- | -- | -- | -- | -- | -- | -- | -- | -- | -- | -- | -- | -- | -- | -- | -- | -- | -- | -- | -- | -- | -- | -- | -- | -- |
| -- | -- | -- | -- | -- | -- | -- | -- | -- | -- | -- | -- | -- | -- | -- | -- | -- | -- | -- | -- | -- | -- | -- | -- | -- | -- | -- | -- | -- | -- |
| -- | -- | -- | -- | -- | -- | -- | -- | -- | -- | -- | -- | -- | -- | -- | -- | -- | -- | -- | -- | -- | -- | -- | -- | -- | -- | -- | -- | -- | -- |
| -- | -- | -- | -- | -- | -- | -- | -- | -- | -- | -- | -- | -- | -- | -- | -- | -- | -- | -- | -- | -- | -- | -- | -- | -- | -- | -- | -- | -- | -- |
| -- | -- | -- | -- | -- | -- | -- | -- | -- | -- | -- | -- | -- | -- | -- | -- | -- | -- | -- | -- | -- | -- | -- | -- | -- | -- | -- | -- | -- | -- |
| -- | -- | -- | -- | -- | -- | -- | -- | -- | -- | -- | -- | -- | -- | -- | -- | -- | -- | -- | -- | -- | -- | -- | -- | -- | -- | -- | -- | -- | -- |
| -- | -- | -- | -- | -- | -- | -- | -- | -- | -- | -- | -- | -- | -- | -- | -- | -- | -- | -- | -- | -- | -- | -- | -- | -- | -- | -- | -- | -- | -- |
| -- | -- | -- | -- | -- | -- | -- | -- | -- | -- | -- | -- | -- | -- | -- | -- | -- | -- | -- | -- | -- | -- | -- | -- | -- | -- | -- | -- | -- | -- |
| -- | -- | -- | -- | -- | -- | -- | -- | -- | -- | -- | -- | -- | -- | -- | -- | -- | -- | -- | -- | -- | -- | -- | -- | -- | -- | -- | -- | -- | -- |
| 15 | 5 | 3 | 4 | 1 | 1 | 0 | 0 | 3 | 1 | 2 | 1 | 10 | 1 | 2 | 1 | 3 | 2 | 3 | 3 | 2 | 2 | 3 | 0 | 0 | 0 | 0 | 0 | 0 | 0 |

English, 2. Colored, 1. Country not given, 8.—Total, 108.

